# Analysis of the current status of research on perianal lesions associated with Crohn's disease: a bibliometric and visualization analysis

**DOI:** 10.3389/fsurg.2026.1624488

**Published:** 2026-03-04

**Authors:** Pengfei Zhou, Jingen Lu, Yi Fu, Xi Huang, Yibo Yao, Yanting Sun, Jiawen Wang

**Affiliations:** 1 Anorectal Department, The First Affiliated Hospital of Henan University of CM, Zhengzhou, Henan, China; 2 Coloproctology Department, Longhua Hospital, Shanghai University of Traditional Chinese Medicine, Shanghai, China

**Keywords:** bibliometric, CiteSpace, Crohn's disease, inflammatory bowel disease, research hotspots

## Abstract

**Objective:**

To analyze the characteristics and research hotspots of perianal lesions associated with Crohn's disease (CD) based on the currently published literature.

**Methods:**

We conducted a comprehensive search of all relevant literature on “Crohn's disease” and “Perianal” from the inception of PubMed, Web of Science Core Collection and Scopus database up to December 31, 2024. Using the bibliometric analysis software CiteSpace 6.4. R1, we visualized and scientifically interpreted the authors, countries, and keywords of the included studies.

**Results:**

A total of 4,286 papers were retrieved using the subject terms “Crohn's disease” and “Perianal”. According to exclusion criteria, 2,134 papers were included in the final analysis. These studies involved 130 researchers, 360 research institutions, and 148 keywords. The analysis revealed that the number of publications on CD with perianal lesions has increased annually since 1979, indicating growing attention to this condition. The United States led in the number of publications, with 452 related papers. The most prolific authors were Ye, Byong Duk and Lightner, Amy L, with 21 published papers each on the topic. The top five keywords were Crohn's disease (357 times), Inflammatory Bowel Disease (202 times), Perianal Fistula (110 times), Ulcerative Colitis (81 times), and Perianal Disease (37 times).

**Conclusion:**

Current research on CD with perianal lesions is primarily focused on mesenchymal stem cells, intestinal inflammation, and biologic therapies. However, further comprehensive studies are needed to improve the early diagnosis and treatment of CD, particularly when perianal symptoms are the initial manifestation.

## 1 Introduction

Crohn's disease (CD) is a chronic, non-specific inflammatory bowel disease (IBD) of unknown etiology. As a subtype of IBD, CD is characterized by transmural inflammation that disrupts the integrity of the intestinal wall and anal canal mucosa, frequently leading to perianal complications such as fistulae and abscesses. Among these complications, perianal fistulizing CD (PFCD) is particularly prevalent, with its incidence showing a marked increase in recent years (1). Fistulae account for a significant proportion of CD-related perianal lesions, approximately 50% of which are classified as complex. Complex fistulae pose substantial clinical challenges due to their high recurrence rates, potential for sphincter damage, and risk of fecal incontinence (2). Furthermore, intestinal inflammation and rectal ulceration may induce anal strictures, severely compromising patients' quality of life (3). Beyond fistulae, CD-associated perianal manifestations encompass a spectrum of conditions, including skin tags, hemorrhoids, anal fissures, abscesses, rectovaginal fistulae, anorectal stenosis, and malignant transformations (4).

CD manifesting with initial perianal involvement presents significant diagnostic challenges due to its insidious clinical onset. The nonspecific nature of early perianal lesions frequently leads to underdiagnosis or delayed recognition. Moreover, as CD constitutes the underlying etiology, isolated surgical intervention targeting perianal manifestations often results in disease recurrence and refractory lesions. Repeated procedures may further compromise perianal tissue integrity and obliterate characteristic pathological features, thereby impairing subsequent diagnostic accuracy and therapeutic decision-making. Current limitations in clinical practice highlight an urgent need for reliable predictive tools to differentiate CD-associated perianal pathology from isolated anorectal disorders. However, the absence of rapid, minimally invasive, and precise diagnostic modalities continues to hinder early identification and targeted management. We found through literature review that previous visualization analyses have mainly focused on the current status of surgical treatment for PFCD (5), the application status of biologics (6), the diagnosis and treatment status of pediatric Crohn's disease (7), the application status of ustekinumab (8), and the global research trends in Crohn's disease treatment over the past 20 years (9), hotspots and trends of perianal fistula of Crohn's disease (10), the development of surgical treatment for Crohn's disease in the past 20 years (11). Through this in-depth review, we have clearly found that although there have been many excellent studies that have sorted out the overall field of Crohn's disease, there is still no research specifically focused on the specific, important, and clinically complex subfield of “Perianal Lesions Associated with Crohn's Disease”.

In recent years, research on perianal Crohn's disease (pCD) has significantly advanced. However, there remains a paucity of studies focusing on two critical aspects: clinical prediction of CD initially presenting with perianal lesions, and comprehensive literature synthesis regarding perianal CD. To address these gaps, this study employed bibliometric methods using CiteSpace 6.4. R1 software to analyze PubMed-indexed literature on CD with perianal manifestations. Through visualization and interpretation of contributing authors, country distributions, and keyword networks, this investigation aims to elucidate the evolving landscape of this research field, identify current research hotspots and emerging trends, and potentially facilitate future studies on predictive for perianal involvement in CD.

## 2 Materials and methods

### 2.1 Data and search

A systematic search was conducted in the PubMed database, Web of Science Core Collection and Scopus database using the subject terms “Crohn's disease” and “Perianal”, with publication date restrictions from database inception through December 31, 2024, and limited to English-language articles.

### 2.2 Inclusion and exclusion criteria

Inclusion Criteria: 1. Confirmed as CD through comprehensive evaluation including clinical, laboratory, imaging, endoscopic, and pathological examinations. 2. Research literature on CD related to perianal lesions; 3. Type of literature as clinical studies, case reports and case series.

Exclusion Criteria: 1. Incomplete information; 2. Perianal lesions that are not CD; 3. Perianal lesions due to exogenous factor, 4. Exclude non-research articles such as books and letters.

### 2.3 Parameter settings

Bibliometric analysis was performed using CiteSpace 6.4. R1. The time frame was configured from January 1, 1961 (the earliest available publication date) to December 31, 2024, with 1-year time slices. Default parameters were maintained for all other settings. Co-occurrence analyses were conducted for three node types: authors, countries, and keywords. Select “Top N” as the ’selection criteria' and set it to 50. Do not perform graph clipping to avoid important information being overlooked. The remaining parameters on the main interface remain at their default settings.

## 3 Result

### 3.1 Literature screening

**Figure 1:**
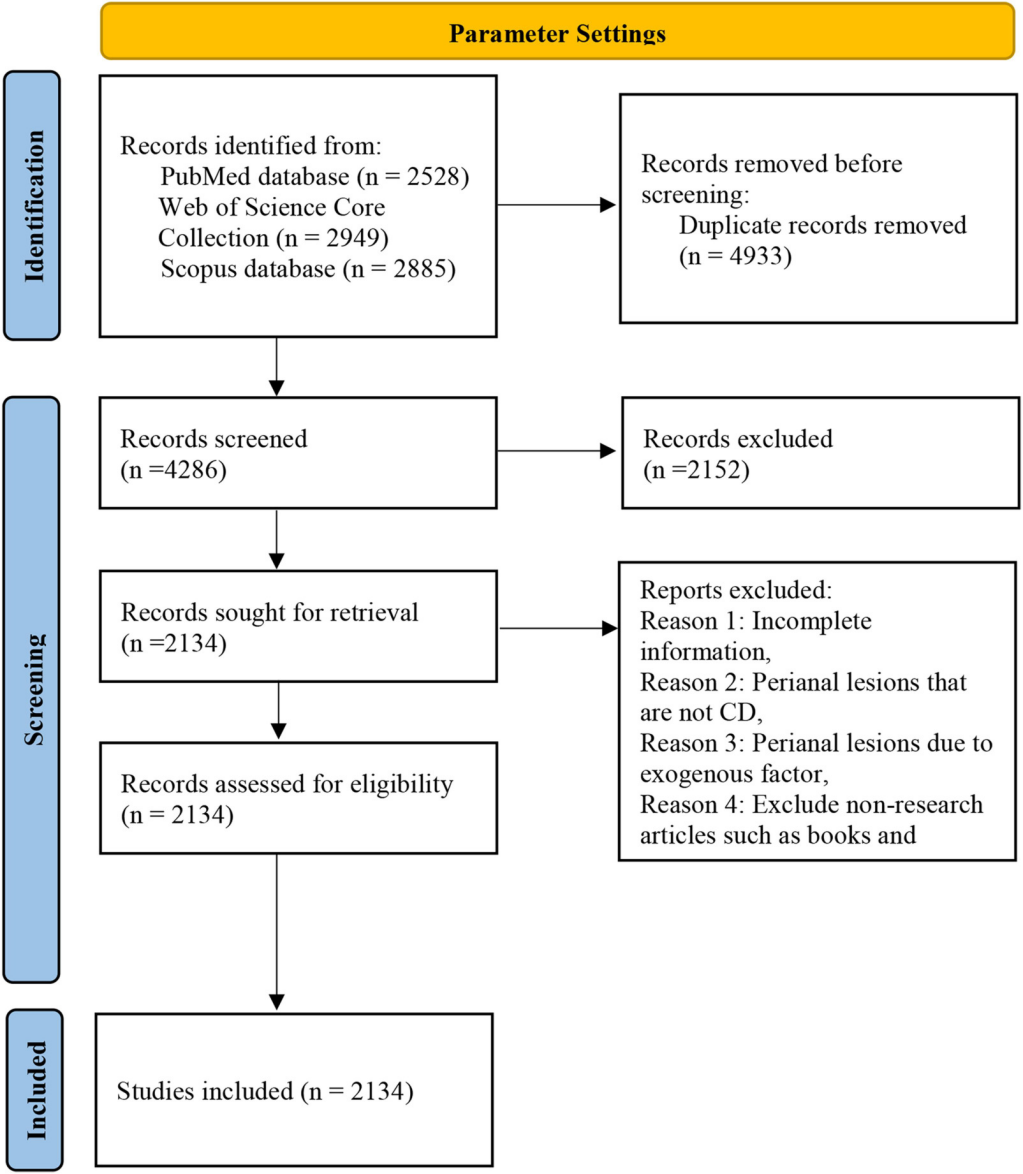
Literature screening.

The PubMed database was searched and a total of 2,528 publications were obtained, 2,949 results from Web of Science Core Collection and 2,885 results from Scopus database. A total of 9,219 articles were obtained, and 4,286 results were obtained by merging and removing 4,933 duplicates. After screening according to inclusion and exclusion criteria, the final results were obtained in 2,134 publications. As shown in Figure 1.

### 3.2 Basic distribution of issuances

**Figure 2:**
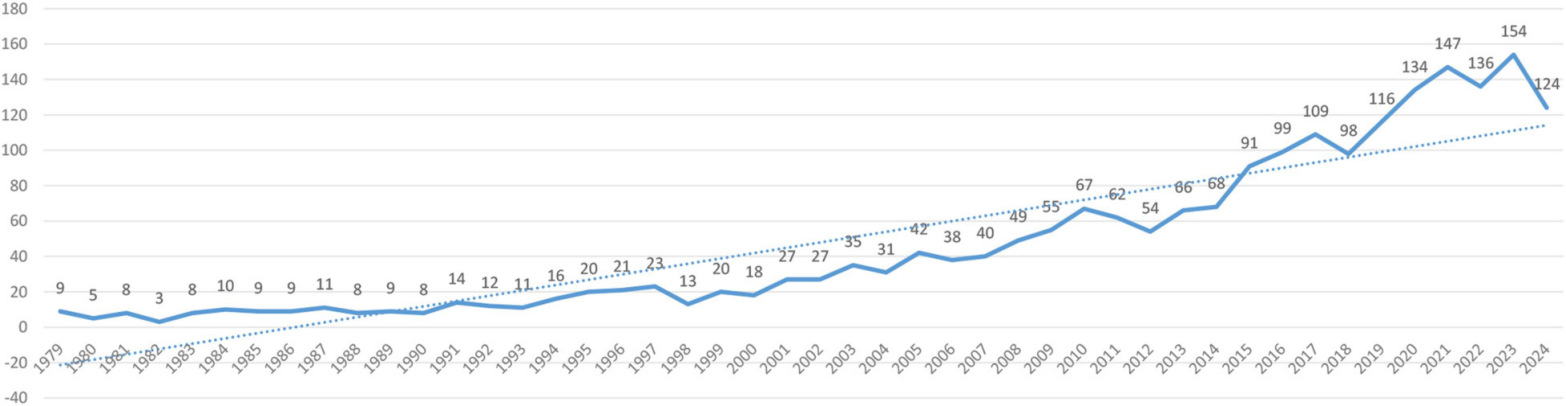
Trends in the number of publications in studies related to Crohn's disease and perianal lesions.

The analysis revealed limited publications on this topic prior to 1978, suggesting minimal research attention during this period. However, a steady year-by-year increase in publications has been observed since 1979, indicating growing scientific interest in CD with perianal lesions (Figure 2).

### 3.3 Country of publication

**Figure 3:**
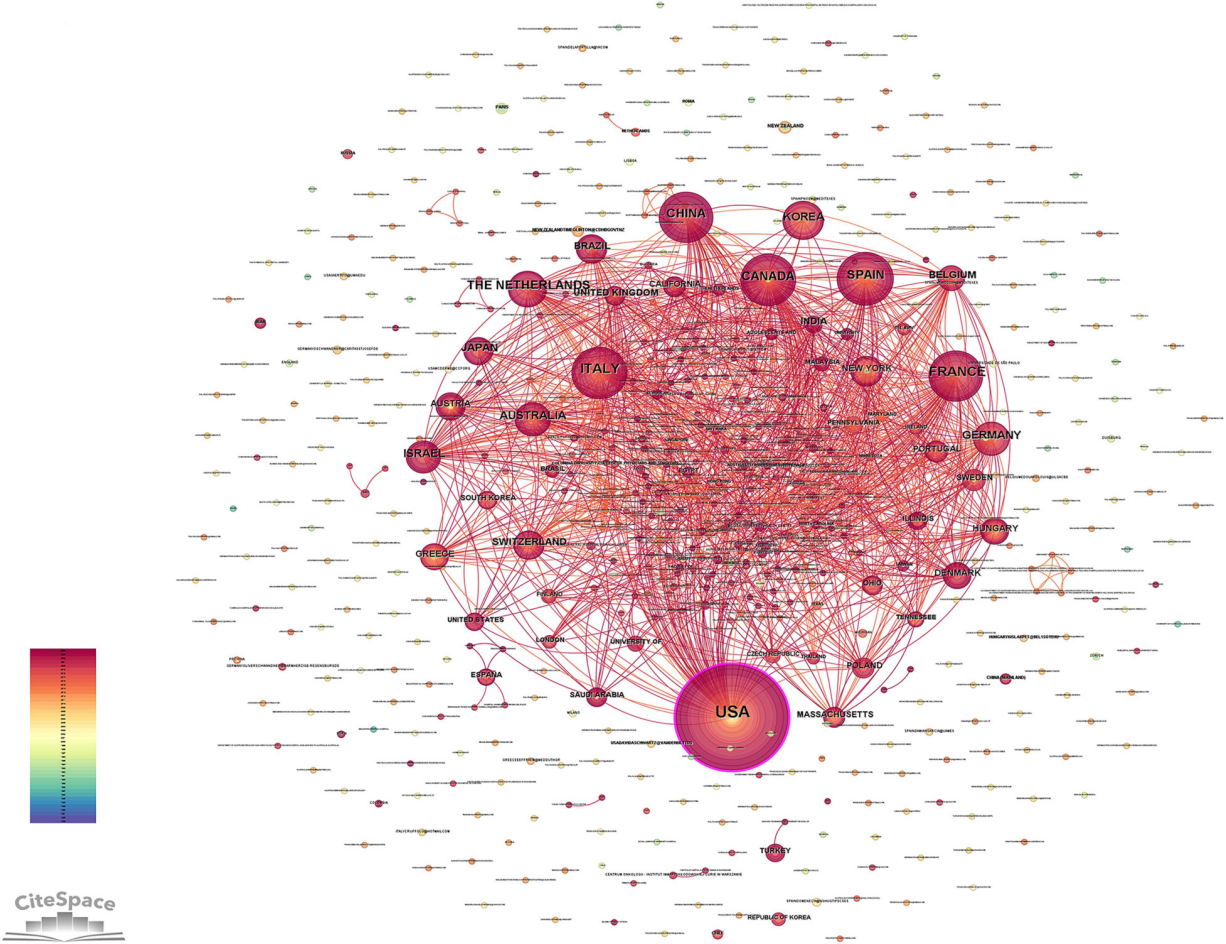
Countries of publications. The United States led in the number of publications, with 452 related papers.

The top 10 countries with the highest number of publications in the research literature on CD with perianal lesions were USA (452), ITALY (119), FRANCE (116), CHINA (108), CANADA (98), SPAIN (95), THE NETHERLANDS (73), KOREA (67) AUSTRALIA (61), GERMANY (58), in the Database. As in Table 1 and Figure 3.

**Table 1:** Top 10 countries in terms of number of publications.

Rank	Country	Volume of publications	Centrality
1	USA	452	0.04
2	Italy	119	0.06
3	France	116	0.04
4	China	108	0.12
5	Canada	98	0.15
6	Spain	95	0.04
7	The Netherlands	73	0.01
8	Korea	67	0.01
9	Australia	61	0.06
10	Germany	58	0.04

### 3.4 Cooperation with the author

**Figure 4:**
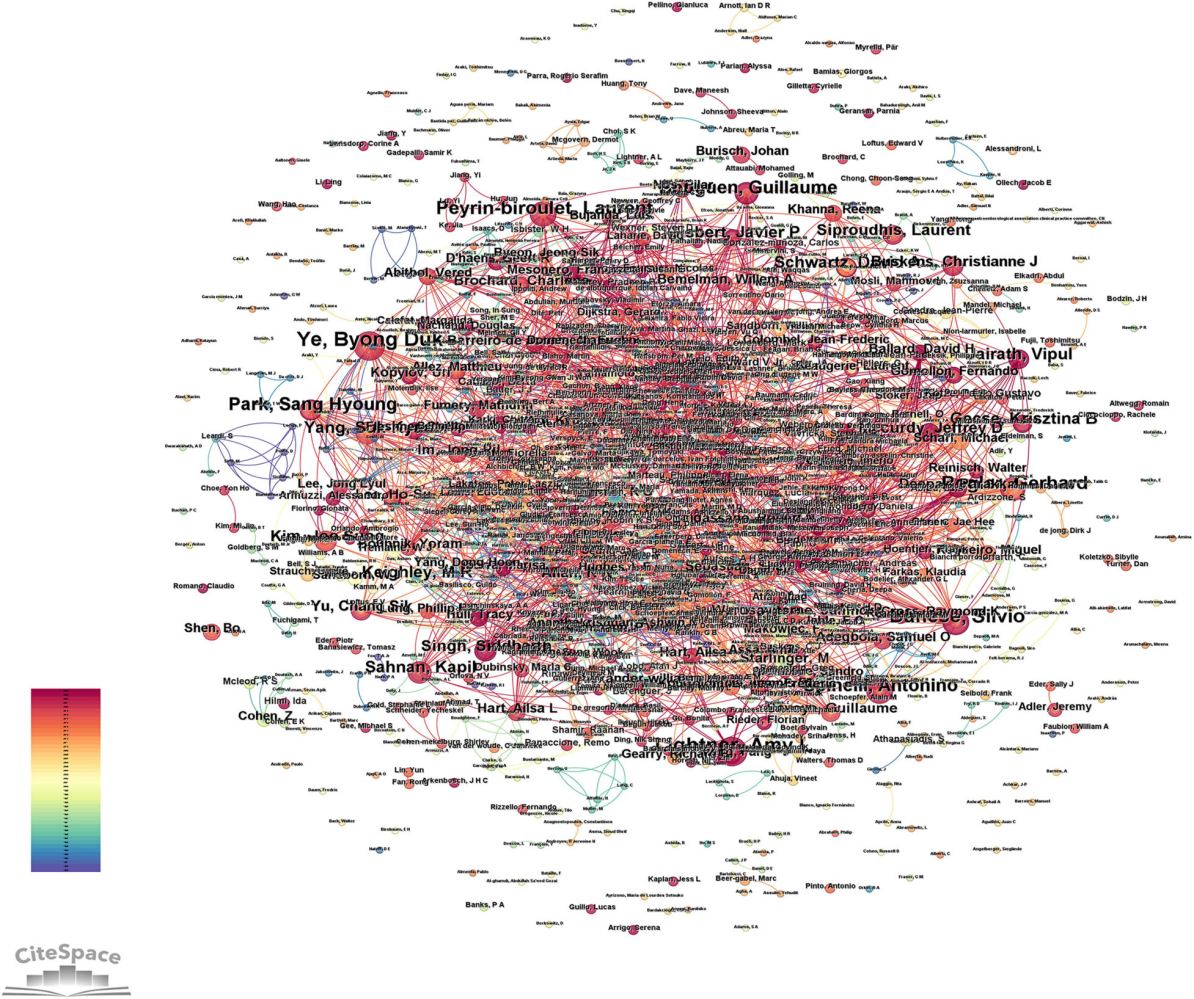
Author collaboration mapping. The most prolific author were Ye, Byong Duk and Lightner, Amy L, with 21 published papers each on the topic.

The analysis constructed an author collaboration network using the included literature, with authors represented as nodes. In the database, this network comprised 1,248 nodes and 2,367 connecting edges, where each node corresponds to an individual author and each edge signifies a collaborative relationship. The thickness of these edges reflects the strength of collaboration, with thicker lines denoting closer or more frequent cooperation between authors. Among the top 10 publications are Ye, Byong Duk (21), Lightner, Amy L (21), Danese, Silvio (18), Peyrin-biroulet, Laurent (17), Rogler, Gerhard (16), Bouguen, Guillaume (15), Spinelli, Antonino (14), Park, Sang Hyoung (14), Gisbert, Javier P (12), Jairath, Vipul (12). As shown in Figure 4.

### 3.5 Keyword Co-occurrence

**Figure 5:**
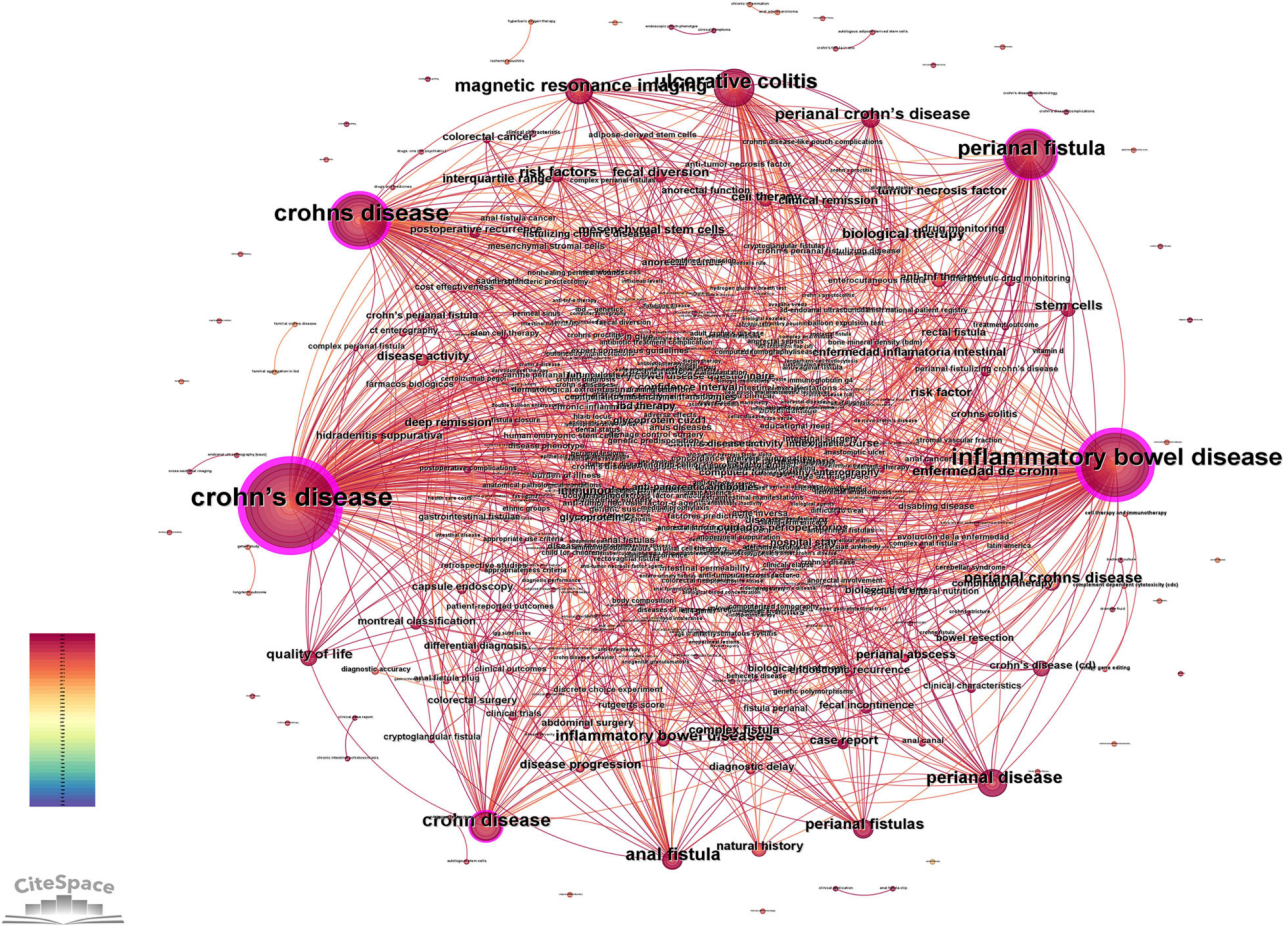
Keyword Co-occurrence mapping. The top five keywords were Crohn's disease (357 times), Inflammatory Bowel Disease (202 times), Perianal Fistula (110 times), Ulcerative Colitis (81 times), and Perianal Disease (37 times).

Keywords encapsulate the core themes of scientific literature, and analyzing high-frequency keywords provides valuable insights into research hotspots and emerging trends within a field. The keyword co-occurrence network derived from the database comprises 464 nodes and 1,135 connecting edges (Figure 5), where each node represents a distinct keyword. Notably, keywords with a centrality score exceeding 0.1 hold greater significance in the study, indicating their pivotal role and substantial influence within the research domain. The top 5 keywords were Crohn's disease (357 times), Inflammatory Bowel Disease (202 times), Perianal Fistula (110 times), Ulcerative Colitis (81 times), Perianal Disease (37times), as in Table 2. The analysis reveals closely related conceptual clusters, with “Crohn's disease” and “Crohn disease” demonstrating similar semantic significance. A hierarchical relationship exists between the broader category of “Inflammatory Bowel Disease” and its specific subtype “Ulcerative Colitis”. Notably, research focus remains predominantly on the general concepts of “Inflammatory Bowel Disease” and “Perianal Fistula” rather than their more specific manifestations in conditions like CD-associated perianal fistulae.

**Table 2:** Top 5 keywords.

Rank	Keyword	Frequency	Centrality
1	Crohn's disease	357	0.43
2	Inflammatory bowel disease	202	0.36
3	Perianal fistula	110	0.24
4	Ulcerative colitis	81	0.21
5	Perianal disease	37	0.11

### 3.6 Keyword clustering

**Figure 6:**
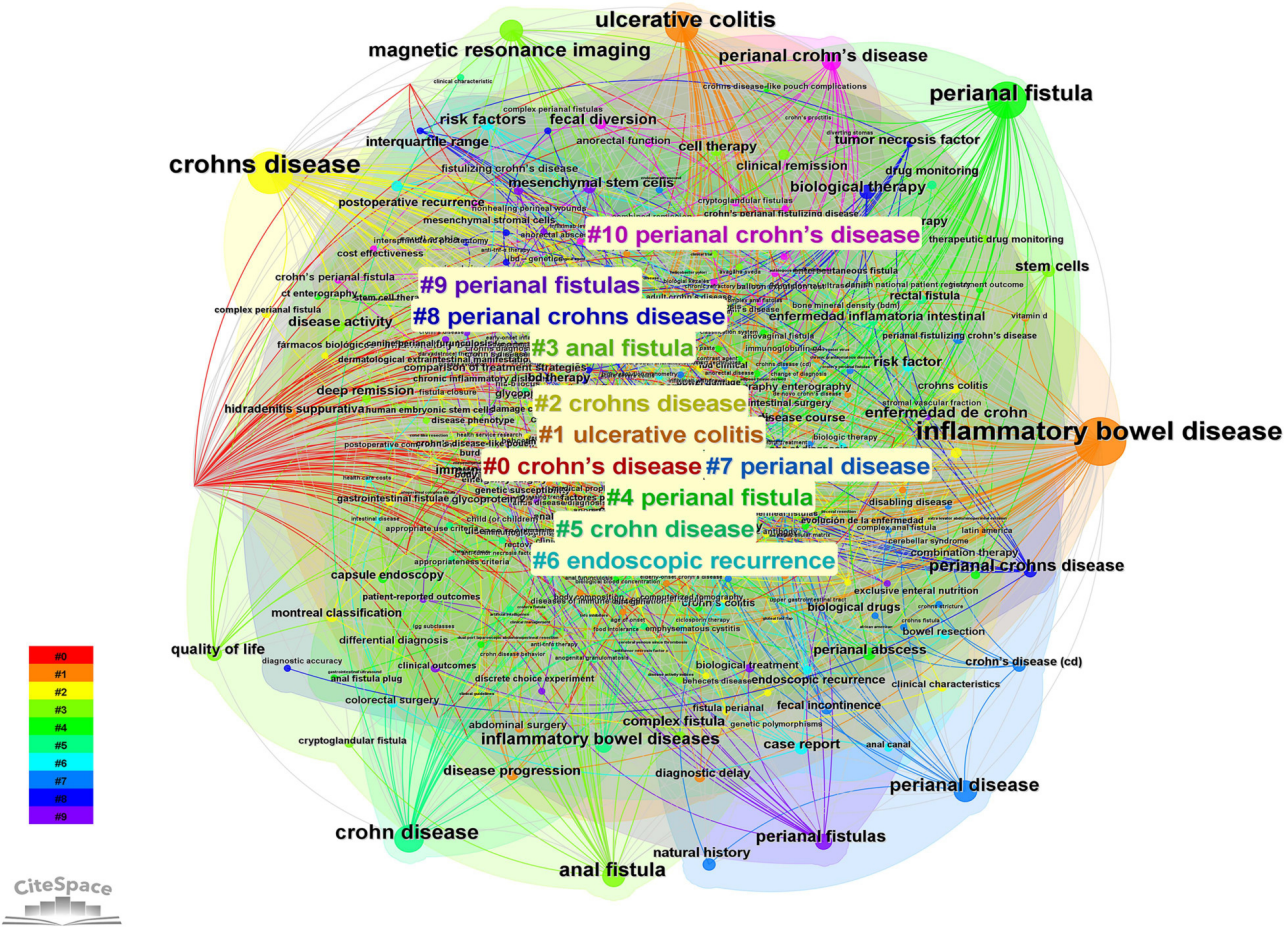
Keyword clustering Map. 10 segments obtained from the cluster analysis have more overlapping parts, indicating that the correlation of each segment is stronger and more connected.

Cluster analysis was carried out on the basis of keyword co-occurrence, and 10 representative clusters were formed according to the similarity of keywords. Modularity Q = 0.4888 > 0.3, indicating that the clustering structure is significant. Mean silhouette = 0.8154 > 0.7, indicating that the clusters have a strong rationality. Figure 6 shows that the 10 segments obtained from the cluster analysis have more overlapping parts, indicating that the correlation of each segment is stronger and more connected, and the labels #2 and #5, #3 and #4 and #7 and #9, #8 and #10, have similar meanings, which also reflects the richness of the theme.

### 3.7 Keyword timeline chart

**Figure 7:**
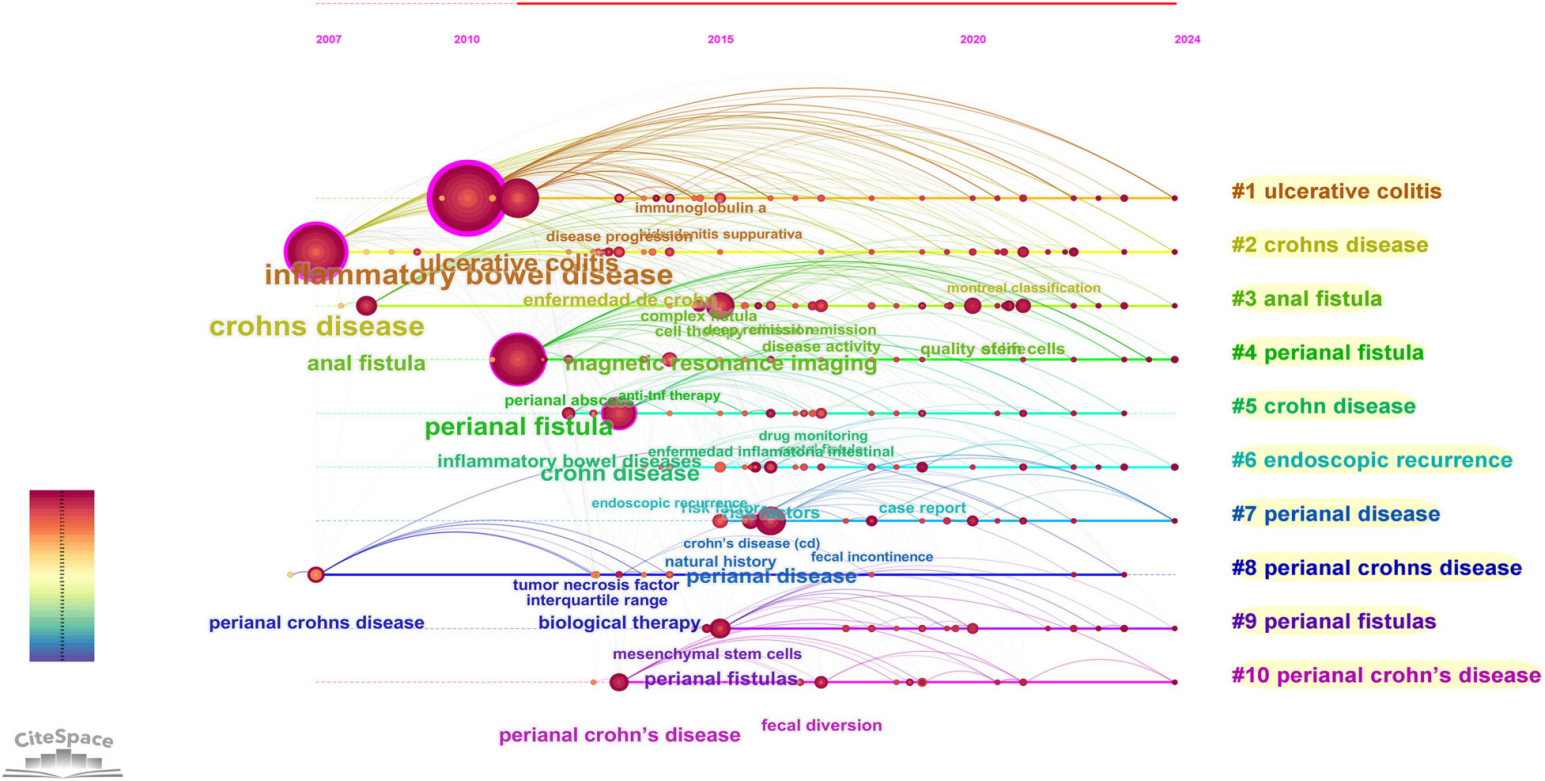
Keyword timeline mapping.

The keyword timeline visualization, constructed through cluster analysis, primarily illustrates the dynamic relationships among different research themes over time. As shown in Figure 7, the vertical axis displays the 10 thematic clusters identified through analysis, while the horizontal axis represents chronological progression. Within this framework, node size corresponds to keyword frequency, node position indicates the year of keyword emergence, and connecting arcs visualize co-occurrence relationships between keywords across different time periods.

### 3.8 Keywords with citation bursts

**Figure 8:**
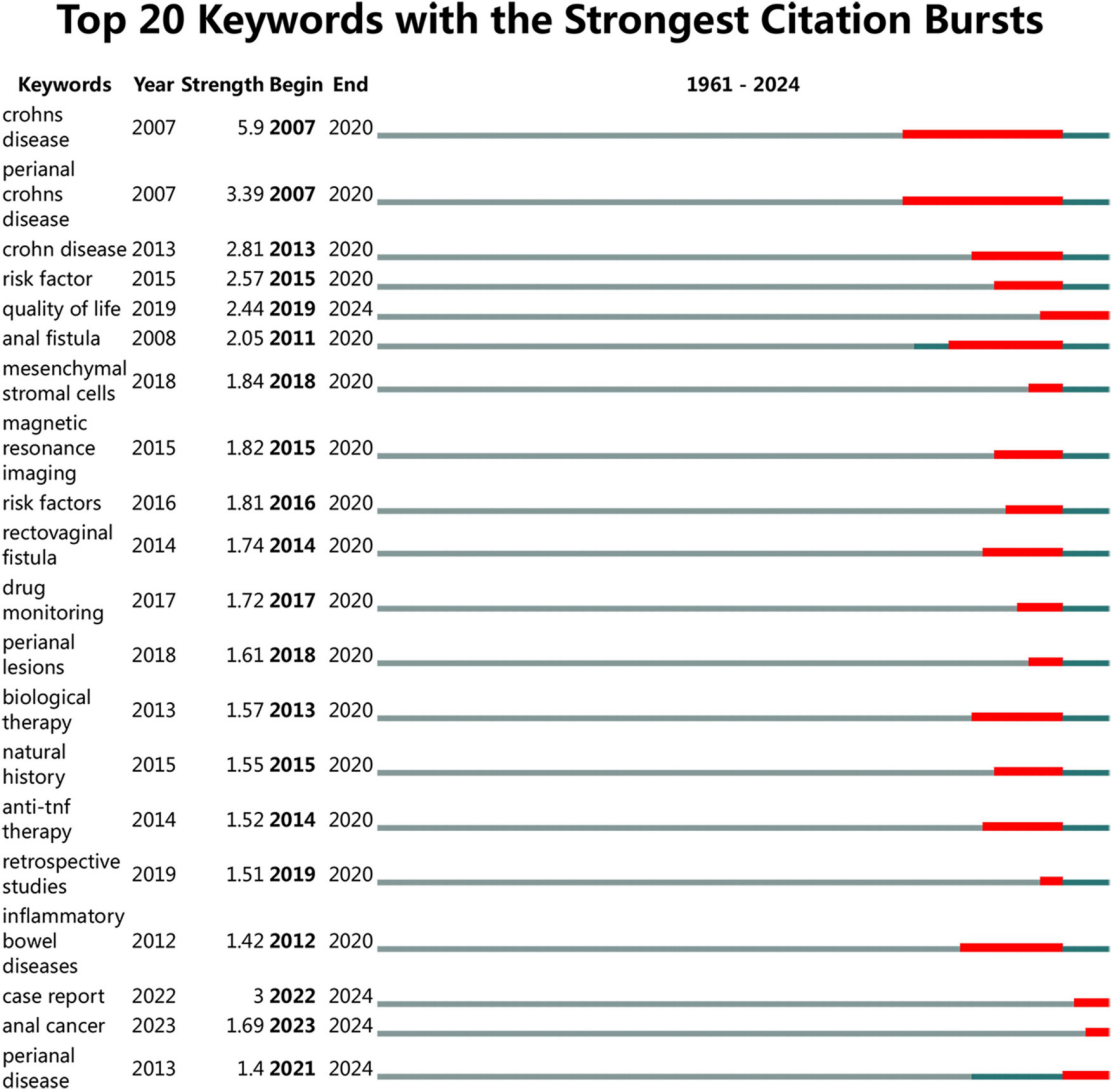
Keywords with strongest citation bursts.

Keyword emergence refers to the rapid increase in attention to specific research topics within a short timeframe, indicating their recognition as academic hotspots. Our visual analysis identified the top 20 emergent keywords, presented in Figure 8. The results demonstrate that “CD perianal fistula” and “Inflammatory Bowel Disease” represent prominent research foci in the database. Recent investigations have increasingly concentrated on several key areas: quality of life assessment, therapeutic drug detection, biologic therapies, anal function impairment, and mesenchymal stromal cell applications. This evolving research landscape reflects both the deepening understanding of CD and emerging future research directions in the field.

## 4 Discussion

CD is a chronic, relapsing systemic inflammatory disorder that primarily involves the gastrointestinal tract, often presenting with extraintestinal manifestations (12). The intestinal pathology of CD is distinguished by discontinuous transmural inflammation of the intestinal wall. Due to its insidious onset and nonspecific early symptoms, CD frequently experiences significant diagnostic delays (13), with approximately 75% of patients receiving confirmation only two years after symptom onset (14). At initial diagnosis, most patients present with inflammatory disease without stenosis or penetrating complications, typically progressing to stricturing or fistulizing phenotypes over time (15). Clinical data indicate that 20%–30% of CD patients demonstrate perianal involvement at first presentation, with cumulative lifetime risk of perianal disease reaching approximately 50% (16). In CD, perianal lesions most frequently manifest as fistulas, though they may also develop in other regions, including the rectovaginal space, bowel-bladder interface, and intra-abdominal structures. While non-fistulizing perianal CD is less common, it remains a clinically significant presentation (17). Notably, perianal symptoms precede the formal diagnosis of CD in approximately 30% of cases (18). Anal fissures and perianal ulcers occur in roughly 17% of patients, with long-term outcomes favoring fissures over ulcers (19). Additionally, anorectal stenosis develops in about 6% of patients within a decade of disease onset (20).

Our analysis of publication trends in the database reveals a consistent increase in research output on CD with perianal lesions since 1979. The annual growth in publications addressing this condition demonstrates its progressively rising significance in the scientific community, reflecting heightened clinical and academic attention over the past four decades. Professor Ye Byong Duk emerged as the most prolific author with the highest publication output, having authored 26 articles. The analysis revealed a total of 348 collaborative publications within this research network, demonstrating strong academic connections among investigators studying the diagnosis and management of CD with perianal lesions. The United States leads in research output on CD with perianal lesions, producing 452 publications, which more than quadruples Italy's output of 119 publications as the second most productive country. This substantial disparity highlights the dominant contribution of U.S. scholars to this field. Given the rising incidence of perianal complications in CD, expanded international research efforts remain crucial to advance understanding and management of this condition. The observed surge in annual publications post-2010 likely mirrors a paradigm shift in PCD management. This period coincides with the widespread adoption of anti-TNFα therapy (particularly adalimumab for maintenance therapy) and the establishment of high-resolution MRI as the gold standard for fistula classification. These diagnostic and therapeutic advances not only expanded the research landscape but also created new clinical questions, thereby fueling a significant volume of investigative work focused on optimizing treatment sequences and assessing therapy response. Keyword clustering analysis identified six representative clusters based on semantic similarity, with modularity Q = 0.488 (exceeding the threshold of 0.3), demonstrating significant cluster structure. The high mean silhouette = 0.8154 (surpassing the 0.7 benchmark) further confirms the robust validity and distinctiveness of these thematic groupings. The cluster analysis yielded six thematic segments with substantial overlap, demonstrating strong interconnections and conceptual correlations among research domains. Keyword emergence analysis revealed two primary research hotspots: “Crohn's disease perianal fistula” and “inflammatory bowel disease”. Notably, recent scholarly attention has shifted toward several emerging frontiers, including quality of life assessment, therapeutic drug monitoring, biologic therapies, anal function preservation, and mesenchymal stromal cell applications. Among them, quality of life is a keyword with a surge in citation from 2019 to 2024. Currently, commonly used quality of life assessment tools include the Inflammatory Bowel Disease Questionnaire (IBDQ), SF-36, EQ-5D, and IBDQ-32 instruments, which better demonstrate the severity of a patient's condition (21). These all evolving research priorities reflect both the maturing understanding of CD pathogenesis and the trajectory of future investigations in this field.

Current research on CD has predominantly focused on mesenchymal stem cells (MSCs) and intestinal inflammation, while investigations into the pathogenesis and therapeutic interventions for perianal lesions remain relatively limited. This trend underscores the potential significance of IBD mechanisms and mesenchymal stem cell biology in driving the development of perianal complications in CD (22). MSCs, primarily isolated from adipose tissue and bone marrow as well as other organ connective tissues (23), have garnered significant therapeutic interest for CD-associated perianal fistulas due to their regenerative capabilities. These cells demonstrate particular efficacy in promoting healing of CD-related perianal lesions (24), especially fistulous tracts that develop from persistent inflammatory processes leading to epithelial defects and ulceration (25). MSCs demonstrate therapeutic potential for CD-associated perianal lesions through their anti-inflammatory and immunomodulatory properties (26). In contrast, conventional pharmacotherapies such as aminosalicylates and corticosteroids show limited efficacy in IBD management, characterized by low remission rates and high disease recurrence (27). Existing evidence further indicates that aminosalicylate therapy provides no significant clinical benefit for luminal CD (28). Scientific investigation into MSCs for therapeutic applications commenced in 2005 (29). The clinical potential of MSCs in treating perianal fistulizing Crohn's disease (pfCD) primarily stems from their unique capacity for multilineage differentiation and targeted tissue engraftment, which enables effective tissue regeneration and repair (30). A systematic review and meta-analysis on MSCs therapy for perianal fistulizing CD demonstrated both safety and clinical efficacy of this treatment approach (31). MSCs possess two fundamental biological properties: multilineage differentiation potential into mesodermal cell types, and potent immunomodulatory effects that suppress immune cell activation, proliferation, differentiation, and maturation processes (32). Our bibliometric analysis clearly captures the translational journey of MSC therapy in PCD. The initial burst of keywords reflects a foundational research phase focused on mechanistic exploration. This was followed by the emergence and citation classic status of pivotal clinical trials, which shifted the intellectual backbone of the field towards clinical efficacy. The subsequent rise of keywords like in recent years signals the field's current maturation, grappling with the challenges of real-world clinical translation. Thus, the bibliometric trends directly map onto the innovation pathway from bench to bedside.

IBD represents a complex, chronic inflammatory disorder with multifactorial pathogenesis involving dysregulated mucosal immunity and intestinal microbiota dysbiosis. In the United States and Europe, IBD affects over 3.5 million individuals (33). Recent epidemiological studies indicate a rising diagnostic incidence of CD, a principal subtype of IBD, within these populations (34). Perianal fistulizing disease (PFD) represents a particularly debilitating manifestation of IBD, significantly compromising patient quality of life (35). Epidemiological data indicate a prevalence of approximately 34% in CD patients compared to 4% in the general IBD population (36). Diagnostic challenges arise when PFD symptoms precede intestinal manifestations, as these cases are frequently not attributed to CD. This clinical scenario often results in delayed diagnosis, with the condition typically receiving proper medical attention only upon symptom recurrence (37). For patients suspected of PFD caused by CD, a comprehensive diagnostic approach incorporating multiple imaging modalities - such as magnetic resonance imaging, intra-anal ultrasound, computed tomography, or dedicated small bowel CT - is recommended to establish a reliable foundation for clinical diagnosis and subsequent treatment planning (38). The clinical manifestations of IBD, including CD, typically feature bloody diarrhea, fever, and intestinal obstruction. Patients with IBD exhibit marked mucosal vulnerability to bleeding, which characteristically affects the entire rectum and colon. CD is distinguished by transmural inflammation that may occur anywhere along the gastrointestinal tract, from the oral cavity to the anus, often demonstrating a discontinuous, “skip lesion” pattern. In contrast, ulcerative colitis typically presents with continuous inflammation extending proximally from the rectum through the colon. However, it is important to note that discontinuous inflammation alone does not definitively confirm a CD diagnosis, as emerging evidence suggests that even prolonged segments of intestinal inflammation may not always be pathognomonic of CD (39). As CD advances, nearly all patients develop intestinal stenosis and varying degrees of perianal lesions. Current therapeutic strategies primarily focus on maintaining clinical remission as the treatment goal, even when clinical symptoms subside while intestinal mucosal inflammation persists. For IBD patients complicated by perianal fistulae, a combined pharmacological and surgical approach is generally recommended, though conservative management may be appropriate for asymptomatic cases or those with well-tolerated fistulae (36). Notably, there remains a significant gap in research comparing the effectiveness of medical vs. surgical interventions when PFD coexists with IBD (40).

Our bibliometric findings have certain implications for clinical practice and scientific research. Firstly, our research provides a reference for clinical doctors, researchers, and decision-makers in the perianal lesions of Crohn's disease. Currently, research on CD mainly focuses on mesenchymal stem cells (MSCs) and intestinal inflammation. This result not only indicates the direction of previous treatments, but also provides a reference for future research. At the same time, it also suggests the need for more teamwork and optimized new methods and ideas in future research to solve this challenging disease. Although our data is included in a large database, there may still be some missing literature, limitations in search strategies, inherent limitations in bibliometric methods, and subjectivity in parameter selection of software such as CiteSpace, which may lead to certain deficiencies in our research. Therefore, in the future, more refined research directions and a larger number of studies need to be included to present the treatment direction of Crohn's disease perianal lesions as clearly as possible.

## 5 Conclusion

While the prevention and treatment of CD have become increasingly standardized, significant research gaps remain, particularly regarding CD cases where perianal symptoms manifest as the initial clinical presentation. Current evidence underscores the need for future investigations to elucidate the underlying pathogenesis of perianal CD lesions and to develop more effective diagnostic and therapeutic strategies. Such research efforts are crucial for improving early detection and intervention in patients presenting with perianal symptoms as their first indication of CD, ultimately enhancing clinical outcomes for this challenging patient population.

## Data Availability

The original contributions presented in the study are included in the article/Supplementary Material, further inquiries can be directed to the corresponding author.
